# Screening of Candidate Leaf Morphology Genes by Integration of QTL Mapping and RNA Sequencing Technologies in Oilseed Rape (*Brassica napus* L.)

**DOI:** 10.1371/journal.pone.0169641

**Published:** 2017-01-09

**Authors:** Hongju Jian, Bo Yang, Aoxiang Zhang, Li Zhang, Xinfu Xu, Jiana Li, Liezhao Liu

**Affiliations:** Chongqing Engineering Research Center for Rapeseed, College of Agronomy and Biotechnology, Southwest University, Beibei, Chongqing, P. R. China; Universidad Miguel Hernández de Elche, SPAIN

## Abstract

Leaf size and shape play important roles in agronomic traits, such as yield, quality and stress responses. Wide variations in leaf morphological traits exist in cultivated varieties of many plant species. By now, the genetics of leaf shape and size have not been characterized in *Brassica napus*. In this study, a population of 172 recombinant inbred lines (RILs) was used for quantitative trait locus (QTL) analysis of leaf morphology traits. Furthermore, fresh young leaves of extreme lines with more leaf lobes (referred to as ‘A’) and extreme lines with fewer lobes (referred to as ‘B’) selected from the RIL population and leaves of dissected lines (referred to as ‘P’) were used for transcriptional analysis. A total of 31 QTLs for the leaf morphological traits tested in this study were identified on 12 chromosomes, explaining 5.32–39.34% of the phenotypic variation. There were 8, 6, 2, 5, 8, and 2 QTLs for PL (petiole length), PN (lobe number), LW (lamina width), LL (Lamina length), LL/LTL (the lamina size ratio) and LTL (leaf total length), respectively. In addition, 74, 1,166 and 1,272 differentially expressed genes (DEGs) were identified in ‘A vs B’, ‘A vs P’ and ‘B vs P’ comparisons, respectively. The Gene ontology (GO) and Kyoto Encyclopedia of Genes and Genomes (KEGG) databases were used to predict the functions of these DEGs. Gene regulators of leaf shape and size, such as *ASYMMETRIC LEAVES 2*, *gibberellin 20-oxidase 3*, genes encoding gibberellin-regulated family protein, genes encoding growth-regulating factor and KNOTTED1-like homeobox were also detected in DEGs. After integrating the QTL mapping and RNA sequencing data, 33 genes, including a gene encoding auxin-responsive GH3 family protein and a gene encoding sphere organelles protein-related gene, were selected as candidates that may control leaf shape. Our findings should be valuable for studies of the genetic control of leaf morphological trait regulation in *B*. *napus*.

## Introduction

Oilseed rape (*Brassica napus* L.) is an important oil crop worldwide that provides both edible oil and industrial materials. Many important traits are controlled by quantitative trait loci (QTLs), such as oil content [1], seed weight [2], flowering time [3], silique length [4] and yield [5]. Meanwhile, some of the candidate genes for these traits have been identified and their functions characterized. Leaf morphology, which has a significant impact on yield, is an important agronomic trait, but information on gene levels in oilseed rape is lacking. Leaves, as lateral organs and determinants of growth, develop from flanking regions of the shoot apical meristem (SAM) [6]. Their morphology involves a balance between cell proliferation and polar cell division and expansion along the proximal–distal, medial–lateral and adaxial–abaxial axes [7–10]. Moreover, plant hormones, such as strigolactone, auxin, cytokinin, and gibberellin (GA), influence leaf development and morphogenesis [11–13].

QTL-based approaches are frequently used to reveal loci that regulate natural phenotypic diversity. QTLs have been identified for leaf morphology in *Brassica rapa* [14, 15], tomato [16], grape [17], oak [18], maize [19], and Arabidopsis [20], suggesting that leaf morphological variation is controlled by multiple genes. QTLs for leaf architecture have also been identified in *Brassica* species [21–24]. Lan and Paterson (2001) [25] identified QTLs, that explained 45% of the phenotypic variation of lamina length in *Brassica oleracea*, and three co-localized with QTLs affecting leaf width (LW). Ten QTLs were later identified that influenced leaf traits using three different mapping populations [21]. Lobe depth and leaf hairiness are controlled by *Brassica rapa gibberellin 20-oxidase 3* (*BrGA20OX3*) and *Brassica rapa glabra1*, which were identified in a synteny analysis of the *Brassica rapa* and *Arabidopsis thaliana* genomes [26, 27]. Leaf hairiness and seed coat color are controlled by the *Brassica rapa transparent testa glabra1* gene, which was successfully cloned [28].

Until now, information on the genetics of leaf morphology in *B*. *napus* has been limited. Here, we used QTL mapping and RNA sequencing (RNA-Seq) to uncover the genetic architecture of leaf morphology in *B*. *napus*. In total, 31 QTLs for leaf morphology traits were identified on 12 chromosomes and differentially expressed genes were identified. These findings will be very useful for studies of the genetic control of leaf morphological trait regulation in *B*. *napus*.

## Materials and Methods

### Plant material, growth conditions and phenotyping

The genetic map constructed by Liu et al. (2013) [29] using an F_9_ RIL mapping population derived from a cross between the parental lines GH06 (female, fewer lobes) and P174 (male, more lobes) was used for QTL mapping in our study. For phenotyping, the parental lines and 172 F_11_ plants were grown in 3 replications from October 2015 to May 2016 in the breeding nursery of Southwest University in Beibei (29°39´N, 106°36´E), Chongqing (China).

**Fig 1:**
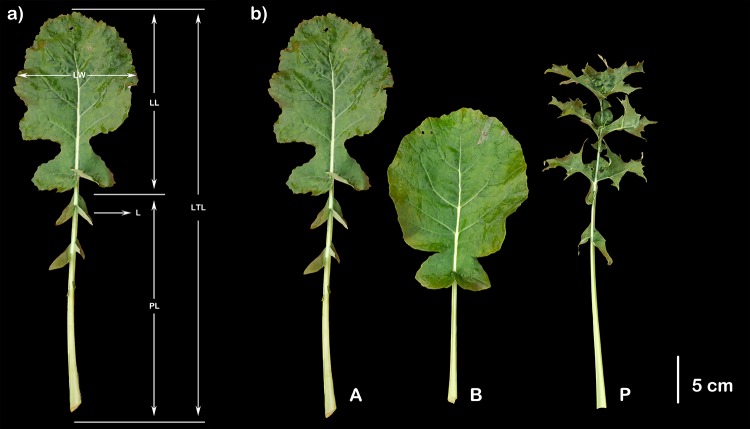
Graphical representation of leaf morphological traits measured in this study (a), extreme lines (b). LL: lamina length; LW: lamina width; PL: petiole length; LTL: leaf total length and L: lobe(s). A: Extreme lines with more lobes; B: extreme lines with fewer lobes; P: dissected lines.

The leaf characteristics were scored on fully developed leaves 60 days after sowing. Lamina length (LL), lamina width (LW), petiole length (PL), leaf total length (LTL), lobe number (PN) and the lamina size ratio (calculated as LL/LTL) were scored and are shown in Fig 1(A). All of the above traits were determined for five plants per line and three leaves per plant. The values of LL, LW, PL and LTL were measured using a ruler as described by Wang et al. (2015) [15]. The SAS 9.0 program (SAS Institute, Inc., Cary, NC, USA) was used for distribution analysis.

### QTL analysis

The linkage map was constructed using 2,795 single-nucleotide polymorphism (SNP) markers with a total map length of 1,832.9 cM and an average distance of 0.66 cM. WinQTL cartographer version 2.5 (http://statgen.ncsu.edu/qtlcart/WQTLCart.htm) was used to screen QTLs for leaf morphological traits by composite interval mapping (CIM). Additionally, 1000 permutations were analyzed to calculate the LOD threshold and the corresponding LOD threshold was used to identify significant QTLs for the corresponding traits. QTL positions were drawn with the software Mapchart [30]. The naming rules for QTLs described by McCouch et al. [31] were used with minor modifications. A QTL designation begins with the QTL and trait abbreviation, such as ‘‘qLL” (q, QTL; LL, lamina length), followed by the linkage group, and finally, the serial number of the QTL in the linkage group (e.g., qLL-A2-1).

### RNA isolation and transcriptome sequencing

The fresh leaves of five lines with more lobes (A) and five lines with fewer lobes (B) were selected from the RIL population, along with leaves from the dissected lines (P), as illustrated in Fig 1(B). These samples were immediately placed in liquid nitrogen and stored at -80°C. Total RNA was extracted using the Plant RNA Mini Kit (TIANGEN, Inc., China). The Agilent 2100 Bioanalyzer (Agilent Technologies, CA, USA) was used to determine the total RNA. qualities. cDNA libraries were constructed and RNA-Seq was performed on an Illumina HiSeq 2000 platform. Paired-end reads of 100-bp were generated for A, B and P samples by Novogene Bioinformatics Technology Co. Ltd. (Beijing, China) according to the manufacturer’s instructions. In addition, sequencing data were uploaded to the NIH Short Read Archive under accession number SRP079113.

### RNA sequencing data analysis

After removing the adapter sequences and low quality sequences from the raw data, clean reads were mapped to the *B*. *napus* reference genome (http://www.genoscope.cns.fr/brassicanapus/) and were then assembled using TopHat 2.0.0 and Cufflinks [32]. The FPKM (fragments per kilobase of exon per million mapped fragments) values were used to estimate the gene expression levels, and differentially expressed genes (DEGs) between two samples were identified with Cuffdiff using the criteria FDR<0.01 and |log_2_ (fold change)|>1. To identify possible transcription factors (TFs) and plant hormone genes, DEGs were aligned to known TFs and plant hormone genes in the *A*. *thaliana* genome that were downloaded from The Plant Transcription Factor Database (http://planttfdb.cbi.pku.edu.cn/index.php) [33] and *Arabidopsis* Hormone Database 2.0 (http://ahd.cbi.pku.edu.cn/) [34], respectively.

### Functional annotation of DEGs

Gene Ontology (GO) term enrichment for all DEGs was analyzed using Goatools (https://github.com/tanghaibao/GOatools), and GO terms were considered significantly enriched if the FDR was less than 0.01. Enriched terms were displayed using the online tool WEGO (Web Gene Ontology annotations Plot, http://wego.genomics.org.cn) [35]. Kyoto Encyclopedia of Genes and Genomes (KEGG) enrichment analysis (hypergeometric test, P-value < 0.01) was performed on all DEGs using the KOBAS2.0 website (http://kobas.cbi.pku.edu.cn/home.do).

### qRT-PCR analysis

To confirm the accuracy of the sequencing data, the expression patterns of 25 randomly selected genes were determined using qRT-PCR analysis. For all three samples, 1 μg of RNA was treated with Turbo DNA-free (Ambion, Austin, TX) and was reverse transcribed into cDNA according to the manufacturer’s instructions (Bio-Rad). Gene-specific primers were designed with Primer3 software (http://frodo.wi.mit.edu/primer3/) and are listed in S1 Table. The 20-μL PCR reactions, each containing 10 μl of SYBR Green Supermix (Bio-Rad), 2.0 μl of cDNA, 0.4 μM each primer, and variable amounts of distilled water, were assembled, and the following cycling program was used for qRT-PCR: 95°C for 30 s; 35 cycles of 95°C for 5 s, followed by annealing at 56–67°C (depending on the primers used) for 30 s. Actin was used as a reference gene to normalize template amounts. The 2^-ΔΔCT^ method was used to estimate the fold change according to Livak & Schmittgen [36]. The data were analyzed using Bio-Rad CFX Manager software. Three biological replicates with three technical replicates were performed for each reaction.

## Results

### Analysis of leaf morphological traits

**Fig 2:**
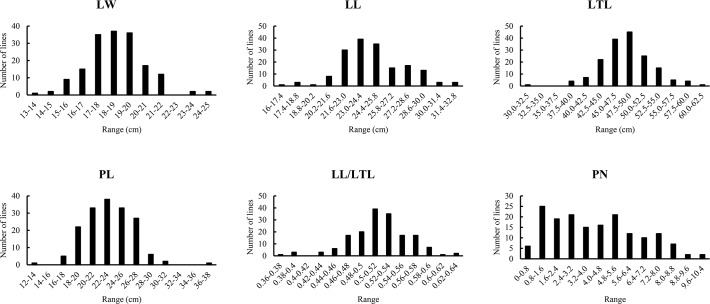
Frequency distributions of leaf morphological traits in RIL lines. LTL: leaf total length (cm); LW: lamina width (cm); LL: lamina length (cm); PL: petiole length (cm); PN: lobe number; LL/LTL: the ratio of lamina width: leaf total length.

The frequency distributions of leaf morphological traits in the RIL population are summarized in Table 1 and Fig 2. We examined a wide range of leaf morphological traits in this study, which showed the transgressive and continuous distributions expected for characters displaying quantitative trait segregation. This observation suggested that there were polygenic effects on leaf morphology and that the data were suitable for QTL analysis (Table 1 and Fig 2).

**Table 1:** Phenotypic variation of leaf morphological traits in RIL lines and their parents. LTL: leaf total length (cm); LW: lamina width (cm); LL: lamina length (cm); PL: petiole length (cm); PN: lobe number; LL/LTL: the ratio of lamina width: leaf total length.

Traits	Parents		RILs		*h*^*2*^
	GH06	P174	Mean	Range	
PN	1.4	8.2	4.2	0.4–10	0.7
LW	23.4	15.8	18.7	13.6–25	0.4
LL	20.2	28.6	24.9	16.1–32.8	0.4
LTL	36.5	58.6	48.3	30.4–60.6	0.6
LL/LTL	0.6	0.5	0.5	0.37–0.62	0.3
PL	18.9	34.3	23.4	12.3–36.1	0.5

*h*^*2*^ is heritability; *h*^*2*^ = (MSv-MSe)/(MSv+(r-1)*MSe)*100%, where MSv is the mean square variation, MSe is the mean square error, and r is the number of replicates.

### Detection of QTLs that influence leaf morphology

**Fig 3:**
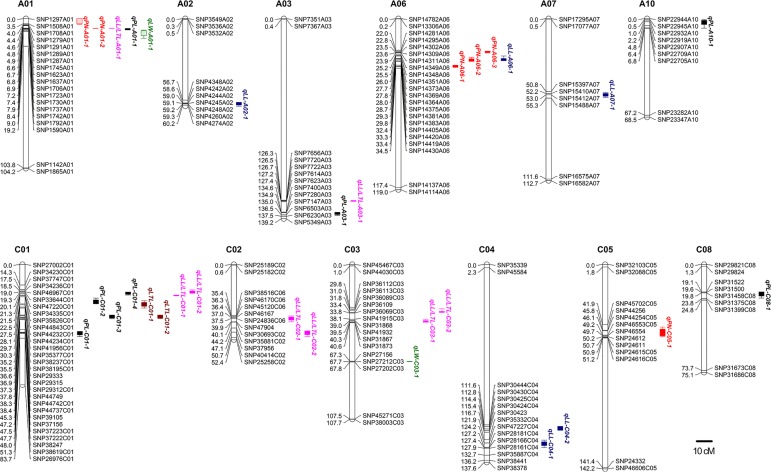
Locations of significant QTLs for leaf morphological traits on the high-density SNP map. For simplicity, only the markers in the QTL confidence intervals, along with the two terminal markers at each end of the QTL-containing chromosomes, are shown. Full map data are provided in Liu et al., 2013. LTL: leaf total length (cm); LW: lamina width (cm); LL: lamina length (cm); PL: petiole length (cm); PN: lobe number; LL/LTL: the ratio of lamina width: leaf total length.

We analyzed leaf morphology traits using a CIM approach. A total of 31 QTLs for leaf morphology traits were identified on 12 chromosomes. These QTLs explained 5.32–39.34% of the phenotypic variation (PV) (Table 2 and Fig 3). There were 8, 6, 2, 5, 8, and 2 QTLs for PL (petiole length), PN (lobe number), LW (lamina width), LL (lamina length), LL/LTL (the lamina size ratio) and LTL (leaf total length), respectively (Table 2).

**Table 2:** Significant QTLs associated with leaf morphological traits in the RIL population. LTL: leaf total length (cm); LW: lamina width (cm); LL: lamina length (cm); PL: petiole length (cm); PN: lobe number; LL/LTL: the ratio of lamina width: leaf total length. a, peak SNP location of the QTL; b, an additive value >0 indicates that additive effects came from GH06, or came from P174 otherwise; c, thresholds values; d, QTL size (cM); e, phenotypic variation.

Trait	Chromosome	Position ^a^ (cM)	Additive ^b^	LOD ^c^	QTL region ^d^	R^2^ (%) ^e^
qPL-A01-1	A01	7.21	2.49	7.06	6.8–7.9	24.94
qPL-A03-1	A03	135.01	-1.09	4.34	134.7–137	6.02
qPL-A10-1	A10	2.21	0.99	4.10	0.5–6.6	8.87
qPL-C01-1	C01	47.51	-0.72	4.79	46.6–50.1	11.20
qPL-C01-2	C01	26.41	-1.28	5.21	23.4–27.4	10.39
qPL-C01-3	C01	35.51	-1.05	5.03	35.2–37.8	9.32
qPL-C01-4	C01	20.41	-1.17	3.60	19.2–21.3	6.90
qPL-C08-1	C08	19.61	-0.98	4.24	19.1–23.4	8.45
qPN-A01-1	A01	1.01	1.49	7.44	0–4	16.04
qPN-A01-2	A01	6.71	1.22	3.41	6.6–6.8	10.94
qPN-A06-1	A06	33.31	-1.22	13.68	32.4–34	24.15
qPN-A06-2	A06	29.31	-1.10	10.16	26.9–29.8	17.28
qPN-A06-3	A06	22.91	-0.85	6.40	22.6–23.9	10.26
qPN-C05-1	C05	48.11	-0.62	4.30	43.6–50.3	6.74
qLW-A01-1	A01	9.01	-0.94	4.15	8–13.7	12.04
qLW-C03-1	C03	67.71	0.98	2.00	67.4–67.7	17.34
qLL-A02-1	A02	59.11	-0.67	3.50	58.2–60.9	7.37
qLL-A06-1	A06	28.01	0.80	4.30	26–29.3	8.15
qLL-A07-1	A07	52.31	0.92	5.60	51.3–54.8	9.76
qLL-C04-1	C04	124.21	0.88	5.65	122.2–127.3	10.49
qLL-C04-2	C04	113.81	0.67	3.06	112.6–115.4	5.58
qLL/LTL-A01-1	A01	6.71	-0.04	7.90	6.6–6.8	39.34
qLL/LTL-A03-1	A03	126.71	0.02	2.03	126.5–127.2	5.32
qLL/LTL-C01-1	C01	21.51	0.02	5.90	21.3–21.9	10.49
qLL/LTL-C01-2	C01	19.31	0.02	6.11	17.7–20.1	9.79
qLL/LTL-C02-1	C02	37.51	0.01	6.02	35.8–39.9	6.14
qLL/LTL-C02-2	C02	47.11	0.01	4.73	46.2–50.1	5.36
qLL/LTL-C03-1	C03	39.51	-0.01	4.86	38.1–40.3	7.43
qLL/LTL-C03-2	C03	32.81	-0.01	3.93	30.9–33.4	6.69
qLTL-C01-1	C01	27.41	-1.57	4.35	26.4–30.3	10.67
qLTL-C01-2	C01	36.51	-1.09	2.79	35.2–37.8	5.60

**PL**: Eight QTLs that were located on five chromosomes explained 86.08% of the PV, and the alleles of six of the QTLs came from P174. *qPL-A01-1*, a major QTL with the largest effect, was detected on chromosome A01 (Fig 3), which accounted for approximately 24.94% of the PV. An allele from GH06 increased PL by 2.49% (additive effect, Table 2). Four QTLs located on chromosome C01 explained 37.81% of the PV, and one QTL located on each of the chromosomes A03, A10 and C08 explained 6.02%–8.87% of the PV (Table 2).

**PN**: There were 2, 3 and 1 QTLs detected on chromosomes A01, A06 and C05, respectively, that explained 85.41% of the PV. A major QTL *qPN-A06-1* explained 24.15% of the PV, and the allele from P174 decreased PN by 1.22% (Table 2).

**LW**: Two QTLs, *qLW-A01-1* and *qLW-C03-1*, explained 12.04% and 17.34% of the PV; their alleles came from P174 and GH06, respectively (Table 2).

**LL**: Five QTLs located on four chromosomes were detected for LL that explained 41.36% of the PV with 5.58%-10.49% of the PV accounted for by a single QTL. The additive effect of *qLL-A02-1* came from P174 (-0.67), and the remaining four QTLs came from the GH06 parental line (Table 2).

**LTL**: Two QTLs for LTL on chromosome C01 were detected; they explained 5.60% and 10.67% of the PV, respectively. Both of them decreased LTL due to a negative additive effect (Table 2). Moreover, the confidence intervals of *qLTL-C01-2* and *qPL-C01-3* overlapped, and the additive effects were both negative, indicating that the loci contributed to both PL and LTL.

**LL/LTL**: Eight QTLs on five chromosomes were detected, explaining 90.56% of the PV with 5.32%-39.34% of the PV accounted for by a single QTL. One major QTL, qLL/LTL-A01-1 on chromosome A01, explained 39.34% of the PV, and the alleles came from P174, as indicated by an additive effect of -0.04 (Table 2). Interestingly, the confidence intervals of *qPN-A01-2* and *qLL/LTL-A01-1* overlapped with a reversed additive effect, suggesting that these loci had pleiotropic effects (Table 2).

### Illumina sequencing and global analysis of gene expression

To obtain a global view of the leaf transcriptome in the different lines, high-throughput RNA-Seq was performed on the three samples (A, B and P) (Fig 1B). In total, 147,360,468 raw reads were generated from the three samples, and more than 96% of the reads remained after low quality and adapter reads were removed, which was sufficient for a quantitative analysis of gene expression. The clean reads were mapped to the reference genome database using TopHat software.

Of the total reads, 74.55%-75.16% were mapped to either multiple (3.59%-4.49%) or unique (70.06%-71.57%) genomic locations, and the remaining 24.84%-25.45% were unmapped (Table 3).

**Table 3:** Summary of read numbers from the RNA-Seq data for the three samples. A: Extreme lines with more lobes; B: extreme lines with fewer lobes; P: dissected lines.

	A	B	P
Total reads	40,256,796	50,814,796	50,589,014
Total mapped	30,255,918	38,159,975	37,714,269
	75.16%	75.10%	74.55%
Multiple mapped	1,444,699	2,019,592	2,269,354
	3.59%	3.97%	4.49%
Uniquely mapped	28,811,219	36,140,383	35444915
	71.57%	71.12%	70.06%
Unmapped	10,000,878	12,654,821	12,874,745
	24.84%	24.90%	25.45%

**Fig 4:**
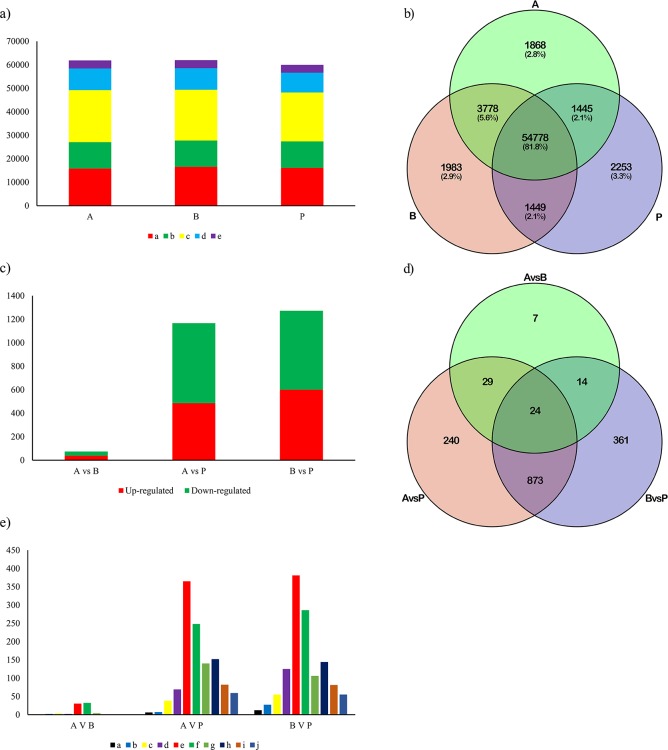
Gene expression profiles and DEGs identified in the three samples. a) Statistical analysis in the three samples, [a: number of genes with very low expression (0<FPKM≤1); b: number of genes with low expression (1<FPKM≤3); c: number of genes with moderate expression (3<FPKM≤15); d: number of genes with high expression (15<FPKM≤60); e: number of genes with very high expression (FPKM>60)] b) Venn Diagram of genes detected in the three samples. c) Identification of DEGs in “A vs B”, “A vs P” and “B vs P”. d) Venn Diagram of DEGs in A vs B, A vs P and B vs P. e) Fold changes in the DEGs detected in “A vs B”, “A vs P” and “B vs P”. [a) Number of genes with a log_2_ fold change≤˗9; b) number of genes with ˗9<log_2_ fold change≤˗7; c) number of genes with ˗7<log_2_ fold change≤˗5; d) number of genes with ˗5<log_2_ fold change≤˗3; e) number of genes with ˗3<log_2_ fold change≤˗1; f) number of genes with 1<log_2_ fold change≤3; g) number of genes with 3<log_2_ fold change≤5; h) number of genes with 3<log_2_ fold change≤5; i) number of genes with 5<log_2_ fold change≤7; j) number of genes with 7<log_2_ fold change≤9] A: Extreme lines with more lobes; B: extreme lines with fewer lobes; P: dissected lines.

In total, 67,554 expressed genes in A, B and P libraries were detected. Additionally, 25.6%-26.9% of genes had FPKM values lower than 1.0, 18.0%-18.8% of genes had FPKM values between 1.0 and 3.0, 34.7%-35.8% of genes had FPKM values between 3.0 and 15.0, 14.0%-14.9% of genes had FPKM values between 15.0 and 60.0, and 5.5%-5.6% of genes had FPKM values more than 60.0 in the three libraries (Fig 4A). A Venn diagram was used to show the distributions of expressed genes in samples A, B and P (Fig 4B). Among these genes, 54,778 (81.1%) were expressed in all three samples; 3,778, 1,449 and 1,445 were co-expressed in A and B, B and P, and A and P, respectively; 1,868, 1,983 and 2,253 genes were unique to A, B and P, respectively.

### Transcriptome changes in the three different types of leaves

To identify the candidate genes responsible for leaf development, we identified the genes that were differentially expressed between each of the samples using the criteria: |log_2_ fold change|>1.0 and FDR<0.01. Between the A and B libraries, 74 DEGs were detected, with 37 up-regulated and 37 down-regulated genes (Fig 4C). In total 1,166 DEGs were detected in the comparison of the A and P libraries, with 485 up-regulated and 681 down-regulated genes, and 1,272 DEGs were identified between the B and P libraries, with 600 up-regulated and 672 down-regulated genes (Fig 4C). Furthermore, 24 DEGs were identified in the “A vs B”, “A vs P” and “B vs P” comparisons, and 53, 38 and 897 were co-expressed in the “A vs B” and “A vs P”, “A vs B” and “B vs P”, “A vs P” and “B vs P” comparisons, respectively. Seven, 240 and 361 genes were specifically expressed in the “A vs B”, “B vs P” and “A vs P” comparisons, respectively (Fig 4D). Moreover, the fold changes (up- or down-regulated) of most DEGs in the “A vs B”, “B vs P” and “A vs P” comparisons were approximately 2–8 (Fig 4E).

### Functional classification of DEGs involved in the development of leaf shape

To determine the important roles of the DEGs described above in the development of leaf shape, GO and KEGG analyses were used to classify their functions. In total, 35, 44 and 44 functional groups were categorized for DEGs from the A vs B, A vs P and B vs P comparisons, respectively (S1 Fig). In each of the three main categories, the terms cell (GO: 0005623), binding (GO: 0005488), and cellular process (GO: 0009987) were dominant. High percentages of the genes were also associated with the terms cell part (GO: 0044464), organelle (GO: 0043226), catalytic activity (GO: 0003824) and metabolic process (GO: 0008152) (S1 Fig). In addition, 4, 5 and 5 KEGG pathways were identified in the A vs B, A vs P and B vs P comparisons, respectively (S2 Table). The top three most enriched pathways assigned to DEGs were Ribosome (ath03010), Carbon metabolism (ath01200) and Carbon fixation in photosynthetic organisms (ath00710).

### Transcription factors and plant hormones involved in leaf morphology

**Fig 5:**
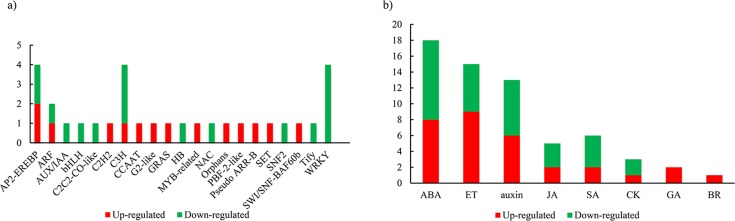
TFs and hormones identified in the DEGs shared between the “A vs P” and “B vs P” comparisons. a) A total of 31 DEGs belong to 21 TF families; b) 63 DEGs are associated with eight types of hormones. A: Extreme lines with more lobes; B: extreme lines with fewer lobes; P: dissected lines.

TFs play key roles in plant organ development [37]. In our study, 31 DEGs belonging to 21 TF families were identified in the DEGs and were shared between the A vs P and B vs P comparisons (Fig 5A). *AUX/IAA* (1), *bHLH* (1), *C*_*2*_*H*_*2*_*-CO-like* (1), *HB* (1), *NAC* (1), *SNF2* (1), *Tify* (1) and *WRKY* (4) were down-regulated, while *C*_*2*_*H*_*2*_, *CCAAT*, *G2-like*, *GRAS*, *MYB-related*, *Orphans*, *PBF-2-like*, *Pseudo ARR-B*, *SET* and *SWI/SNF-BAF60b* were up-regulated. The rest of the TF families, such as *AP2-EREBP*, *ARF* and *C3H*, were either down- or up-regulated.

It is well known that plant hormones, such as auxin, cytokinin (CK), gibberellin (GA), abscisic acid (ABA), ethylene (ET), brassinosteroids (BR), jasmonic acid (JA), and salicylic acid (SA), play vital roles in plant organ development [11, 12]. In this study, 63 hormone-associated DEGs were identified in the DEGs shared between A vs P and B vs P comparisons (Fig 5B). The top three hormones with the most associated DEGs were ABA (18), ET (15) and auxin (13), accounting for 73% of the hormone-associated DEGs. Almost half of the total hormone-associated DEGs were up-regulated, and all of the GA and BR genes were up-regulated.

### Expression analysis of the orthologous genes influencing leaf morphology in *Arabidopsis*

It was unknown whether any of the DEGs were associated with controlling leaf morphology in *B*. *napus*. In total, 201 genes with roles in leaf development were identified in the *B*. *napus* genome based on their corresponding homologs in the *A*. *thaliana* genome. To identify pivotal genes that controlled leaf morphology, RNA-Seq data were filtered using the criteria: |log_2_ fold change|≥1.0 and FPKM ≥1 comparing P lines (for at least one sample). As illustrated in Table 4, 9 *GRF*s (1 *GRF1*, 2 *GRF3*, 1 *GRF4*, 3 *GRF5* and 2 *GRF8*), 7 GA-related genes (2 *GA20OX3* and 5 *gibberellin-regulated family proteins*), 2 *AS2*s and 2 *KNAT*s (1 *KNAT4* and 1 *KNAT5*) were identified. Moreover, 13 of these genes were up-regulated, including 8 *GRF*s, 2 *AS2*, 2 *KNAT*s and 1 *GA*-regulated family protein.

**Table 4:** Expression analysis of the orthologous genes that influence leaf morphology in *Arabidopsis*. A: Extreme lines with more lobes; B: extreme lines with fewer lobes; P: dissected lines.

Gene ID	A-FPKM	B-FPKM	P-FPKM	AGI ID	Function description
BnaA02g12180D	5.07	7.72	2.94	AT1G65620	ASYMMETRIC LEAVES 2 (AS2)
BnaCnng14500D	5.93	5.15	2.60	AT1G65620	ASYMMETRIC LEAVES 2 (AS2)
BnaC09g48370D	0.92	1.10	5.22	AT5G07200	gibberellin 20-oxidase 3 (GA20OX3)
BnaA10g23640D	3.69	1.79	7.30	AT5G07200	gibberellin 20-oxidase 3 (GA20OX3)
BnaA09g30450D	5.43	6.76	17.56	AT1G22690	Gibberellin-regulated family protein
BnaCnng22380D	2.41	0.16	16.92	AT2G14900	Gibberellin-regulated family protein
BnaA01g31950D	6.06	4.04	14.74	AT1G74670	Gibberellin-regulated family protein
BnaA07g03270D	3.05	4.72	6.65	AT5G14920	Gibberellin-regulated family protein
BnaC03g45610D	4.30	6.83	2.79	AT2G14900	Gibberellin-regulated family protein
BnaC03g27220D	10.37	10.94	5.43	AT2G22840	growth-regulating factor 1 (GRF1)
BnaA03g16700D	4.86	4.06	2.19	AT2G36400	growth-regulating factor 3 (GRF3)
BnaA05g07850D	2.21	2.66	0.64	AT2G36400	growth-regulating factor 3 (GRF3)
BnaC08g24080D	17.99	18.09	8.97	AT3G52910	growth-regulating factor 4 (GRF4)
BnaC08g28950D	0.28	0.18	2.14	AT3G13960	growth-regulating factor 5 (GRF5)
BnaA05g25480D	3.15	4.07	1.40	AT3G13960	growth-regulating factor 5 (GRF5)
BnaA03g33180D	2.45	2.61	0.77	AT3G13960	growth-regulating factor 5 (GRF5)
BnaA03g46480D	4.64	2.96	1.82	AT4G24150	growth-regulating factor 8 (GRF8)
BnaC07g38750D	2.79	2.35	0.60	AT4G24150	growth-regulating factor 8 (GRF8)
BnaCnng20070D	2.83	1.68	0.00	AT5G11060	KNOTTED1-like homeobox gene 4 (KNAT4)
BnaA01g04870D	4.26	4.09	1.92	AT4G32040	KNOTTED1-like homeobox gene 5 (KNAT5)

### Screening for candidate genes that control leaf morphology by the integration of QTL mapping and RNA sequencing

In total, 1,205 genes were identified in QTL regions by aligning SNP marker physical locations to the oilseed reference genome. The expression profiles of these genes in the three samples were determined via RNA-Seq (S4 Table). Thirty-three candidate genes (21 up-regulated and 12 down-regulated) were identified using the criteria: |log_2_ fold change|≥1.0 and FPKM ≥1 comparing B samples (for at least one sample). These genes included two encoding auxin-responsive GH3 family proteins (*BnaA06g06420D* and *BnaA06g06400D*), the *sphere organelles protein-related* gene (*BnaA06g07690D*), the ARF-GAP domain 4 (*BnaA06g06680D*), and two clathrin-encoding genes (*BnaA01g31000D* and *BnaA01g31010D*) (Table 5).

**Table 5:** Expression analysis of candidate genes for the regulation of leaf development identified using QTL and RNA-Seq analyses. A: Extreme lines with more lobes; B: extreme lines with fewer lobes; P: dissected lines.

Gene ID	A-FPKM	B-FPKM	Log2(A-FPKM/B-FPKM)	AGI ID	Function description
BnaA01g31000D	8.2	3.6	1.2	AT3G11130	Clathrin, heavy chain
BnaA01g31010D	25.5	12.2	1.1	AT3G11130	Clathrin, heavy chain
BnaA01g31870D	0.3	1.0	-1.8	AT3G10700	Galacturonic acid kinase (GalAK)
BnaA02g01890D	0.7	1.6	-1.1	AT5G13820	Telomeric DNA binding protein 1 (TBP1)
BnaA03g05340D	2.3	0.6	1.9	AT5G15265	unknown protein
BnaA03g06280D	2.2	1.1	1.0	AT5G16870	Peptidyl-tRNA hydrolase II (PTH2) family protein
BnaA03g11330D	0.7	1.4	-1.1	AT5G55550	RNA-binding (RRM/RBD/RNP motifs) family protein
BnaA03g11350D	10.0	3.8	1.4	AT5G55510	Mitochondrial import inner membrane translocase subunit Tim17/Tim22/Tim23 family protein
BnaA06g05590D	82.5	188.1	-1.2	AT1G09690	Translation protein SH3-like family protein
BnaA06g06360D	67.8	28.1	1.3	AT1G10522	unknown protein
BnaA06g06400D	0.1	1.2	-3.4	AT1G48660	Auxin-responsive GH3 family protein
BnaA06g06420D	15.5	4.9	1.7	AT5G51470	Auxin-responsive GH3 family protein
BnaA06g06510D	13.9	6.1	1.2	AT1G10640	Pectin lyase-like superfamily protein
BnaA06g06680D	5.6	2.7	1.1	AT1G10870	ARF-GAP domain 4 (AGD4)
BnaA06g06840D	3.9	1.1	1.8	AT1G11112	unknown protein
BnaA06g06880D	0.1	3.4	-5.1	AT1G11170	Protein of unknown function (DUF707)
BnaA06g07330D	1.2	0.3	2.0	AT1G11740	Ankyrin repeat family protein
BnaA06g07410D	31.2	4.4	2.8	AT1G13360	unknown protein
BnaA06g07560D	0.8	1.7	-1.1	AT1G13180	DISTORTED TRICHOMES 1 (DIS1)
BnaA06g07670D	1.0	0.3	2.0	AT1G13040	Pentatricopeptide repeat (PPR-like) superfamily protein
BnaA06g07690D	4.7	1.1	2.2	AT1G13030	Sphere organelle protein-related
BnaA07g17610D	0.6	2.2	-1.9	AT3G58130	N-acetylglucosaminylphosphatidylinositol de-N-acetylase family protein
BnaA07g18100D	1.1	0.3	2.1	AT3G58820	F-box/RNI-like superfamily protein
BnaA07g18370D	2.5	1.0	1.4	AT3G59470	Far-red impaired responsive (FAR1) family protein
BnaA10g25600D	2.3	1.2	1.0	AT5G04610	S-adenosyl-L-methionine-dependent methyltransferases superfamily protein
BnaA10g25870D	0.4	1.9	-2.3	AT5G04340	Zinc finger of *Arabidopsis thaliana* 6 (ZAT6)
BnaC01g15860D	1.9	0.9	1.1	AT4G24026	unknown protein
BnaC01g15960D	14.8	5.5	1.4	AT4G24130	Protein of unknown function, DUF538
BnaC01g16160D	3.5	8.5	-1.3	AT4G24265	unknown protein
BnaC01g29560D	12.6	4.9	1.4	AT1G61580	R-protein L3 B (RPL3B)
BnaC01g29590D	1.8	0.6	1.7	AT1G52950	Nucleic acid-binding, OB-fold-like protein
BnaC01g29740D	0.5	1.3	-1.3	AT3G55580	Regulator of chromosome condensation (RCC1) family protein
BnaC08g27570D	1.3	2.6	-1.0	AT3G56700	Fatty acid reductase 6 (FAR6)

### Verification of transcriptome sequencing

**Fig 6:**
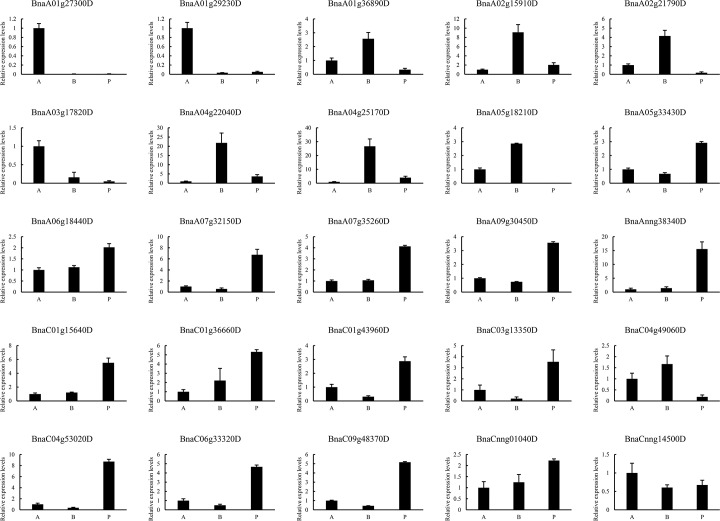
qRT-PCR validation of the expression patterns of 25 randomly selected genes identified in transcriptome sequencing. A: Extreme lines with more lobes; B: extreme lines with fewer lobes; P: dissected lines.

To confirm the results of RNA sequencing, 25 DEGs were selected randomly for qRT-PCR analysis (Fig 6). All DEGs exhibited similar expression patterns supporting the reliability of the data from RNA sequencing.

## Discussion

In our study, QTL and RNA-Seq analyses were used to screen for candidate genes that could influence leaf morphology in *B*. *napus*. We identified 31 QTLs for leaf morphological traits that were located on 12 chromosomes. One major QTL, located on chromosome A01, controlled PL and LL/LTL (Table 2). Until now, no QTL mapping studies of leaf morphological traits in *B*. *napus* have been reported. Leaf morphology shows extensive variation among different plant species and even among varieties of the same species and is strongly influenced by genetics. Experiments in model species have identified some of the genes and mechanisms controlling leaf size and shape [7, 8].

Screening of our QTL and RNA-Seq results, we identified 33 candidate genes, and the expression profiles of 25 genes were verified by qRT-PCR. Unfortunately, no evidence that these genes regulated leaf size and shape has been previously reported. Further studies, especially those focusing on *BnaA06g07690D* (*sphere organelles protein-related*), *BnaA06g06420D* (Auxin-responsive GH3 family protein) and *BnaA06g06680D* (*ARF-GAP domain 4*), should be conducted to confirm the roles of these genes in leaf development.

To understand the molecular mechanisms controlling the final size and shape of *B*. *napus* leaves, we used RNA-Seq to measure the expression levels of the *B*. *napus* homologs of these potential regulators in three samples with different leaf shapes. There were 2, 2, 5, 9, 1 and 1 homologs for *AS2*, *GA20OX3*, gibberellin-regulated family protein, growth-regulating factor, *KNOTTED1-like homeobox gene 4* (*KNAT4*), and *KNOTTED1-like homeobox gene 5* (*KNAT5*), respectively (Table 4). Coordinated cell proliferation and cell expansion are the main types of growth affecting leaf size and shape [38]. In model systems such as *A*. *thaliana*, several genes that control leaf growth have been identified. These genes determine leaf size and shape by regulating the balance between cell proliferation and cell expansion.

In our study, both *AS2* and *KNOX1* genes were down-regulated in the P sample compared with their expression in the A or B samples. *BnaCnng20070D*, which is homologous to *AT5G11060* (*KNAT4*), and *BnaA01g04870D*, which is homologous to *AT4G32040* (*KNAT5*), are *KNOTTED1*-like homeobox genes with important roles in promoting leaf complexity [39]. *KNOX1* genes are expressed in the SAM and maintain indeterminacy of leaf stem cells [40]. *KNOX1* genes also play pivotal roles in leaf initiation in most plants with compound leaves. Their influence on leaf development has been confirmed by altering their expression profiles [41–44]. AS2, a key negative regulator of *KNOX* genes, is expressed in leaves [45]. Defects in leaf patterning have been detected in *as2*, whereas in *AS2-*overexpressing lines, lamina outgrowths occurred on the abaxial side of the leaf.

There are 36 homologs of the nine *AtGRF*s in *B*. *napus*. In this study, nine *GRF*s, including three *GRF5*s (homologous genes of *AT3G13960*), were identified. *BnaC08g28950D* was up-regulated while *BnaA05g25480D* and *BnaA03g33180D* were down-regulated in the P sample compared with the A or B samples. Previous studies have indicated that *AtGRF5* plays important roles in leaf cell proliferation [46]. Another gene, *ANGUSTIFOLIA3* (*AN3*), works together with *AtGRF5* and is involved in leaf cell proliferation, leaf size and shape regulation [47]. The double mutants of *an3* and *atgrf5* exhibited narrow-leaf phenotypes because of reduced cell numbers. By contrast, cell number increased and leaves grew larger than the wild type in *AN3-* or *AtGRF5*-overexpressing lines. These results indicated that functional differentiation occurred in the homologous *GRF5* genes.

Moreover, plant hormones, such as GAs, interact with KNOX pathways, influencing leaf shape and final size [13]. In this study, *BnaC09g48370D* and *BnaA10g23640D*, homologs of *AT5G07200* (*GIBBERELLIN 20 OXIDASE 3*), were identified and were up-regulated in P samples compared with A or B samples. Furthermore, five gibberellin-regulated family proteins were also identified, four of which were up-regulated in P samples compared with A or B samples. The GA-biosynthesis gene GA 20-oxidase [48] is suppressed by the *Nicotiana tabacum* Homeobox15 (*NTH15*) gene, and GA levels subsequently decreased in tobacco leaves of *NTH15*-overexpressing lines [49]. Previous studies have also shown that GA synthesis is regulated by *KNOX* genes in *Arabidopsis*. The altered leaf shape phenotype is partially alleviated by increasing GA in *Arabidopsis* plants through the ectopic expression of *KNOX* genes in the leaves [50]. Li et al., [26] also identified a QTL for leaf lobe and leaf hairiness containing *GIBBERELLIN 20 OXIDASE 3*.

## Conclusions

In summary, a total of 31 QTLs for the leaf morphological traits evaluated in this study were identified on 12 chromosomes, explaining 5.32–39.34% of the phenotypic variation using a population of 172 RILs. In addition, 74, 1,166 and 1,272 DEGs were identified using RNA-Seq technologies in the ‘A vs B’, ‘A vs P’ and ‘B vs P’ comparisons, respectively. In addition, 1,205 genes were identified in QTL regions by aligning the SNP marker physical locations with the oilseed reference genome. Thirty-three candidate genes were identified by QTL mapping and RNA-Seq technologies. Our findings should be valuable for studies of the genetic control of the regulation of leaf morphological traits in *B*. *napus*.

## Supporting Information

S1 Fig GO categories of DEGs for “A vs B”, “A vs P” and “B vs P”. (TIF)

S1 Table Primers used for qRT-PCR analysis. (XLSX)

S2 Table Significantly enriched pathways among DEGs for “A vs B”, “A vs P” and “B vs P”. A: Extreme lines with more lobes; B: extreme lines with fewer lobes; P: dissected lines.
(XLSX)

S3 Table Common DEGs between “A vs P” and “B vs P”. (XLSX)

S4 Table Expression profiles of 1,205 genes identified in the QTL regions of the three samples. (XLSX)
