# Supplementary figures and images for: Screening of Candidate Leaf Morphology Genes by Integration of QTL Mapping and RNA Sequencing Technologies in Oilseed Rape (Brassica napus L.)
Source: PLoS One. 2017 Jan 9;12(1):e0169641. doi: 10.1371/journal.pone.0169641 (PMC5222374; doi:10.1371/journal.pone.0169641)

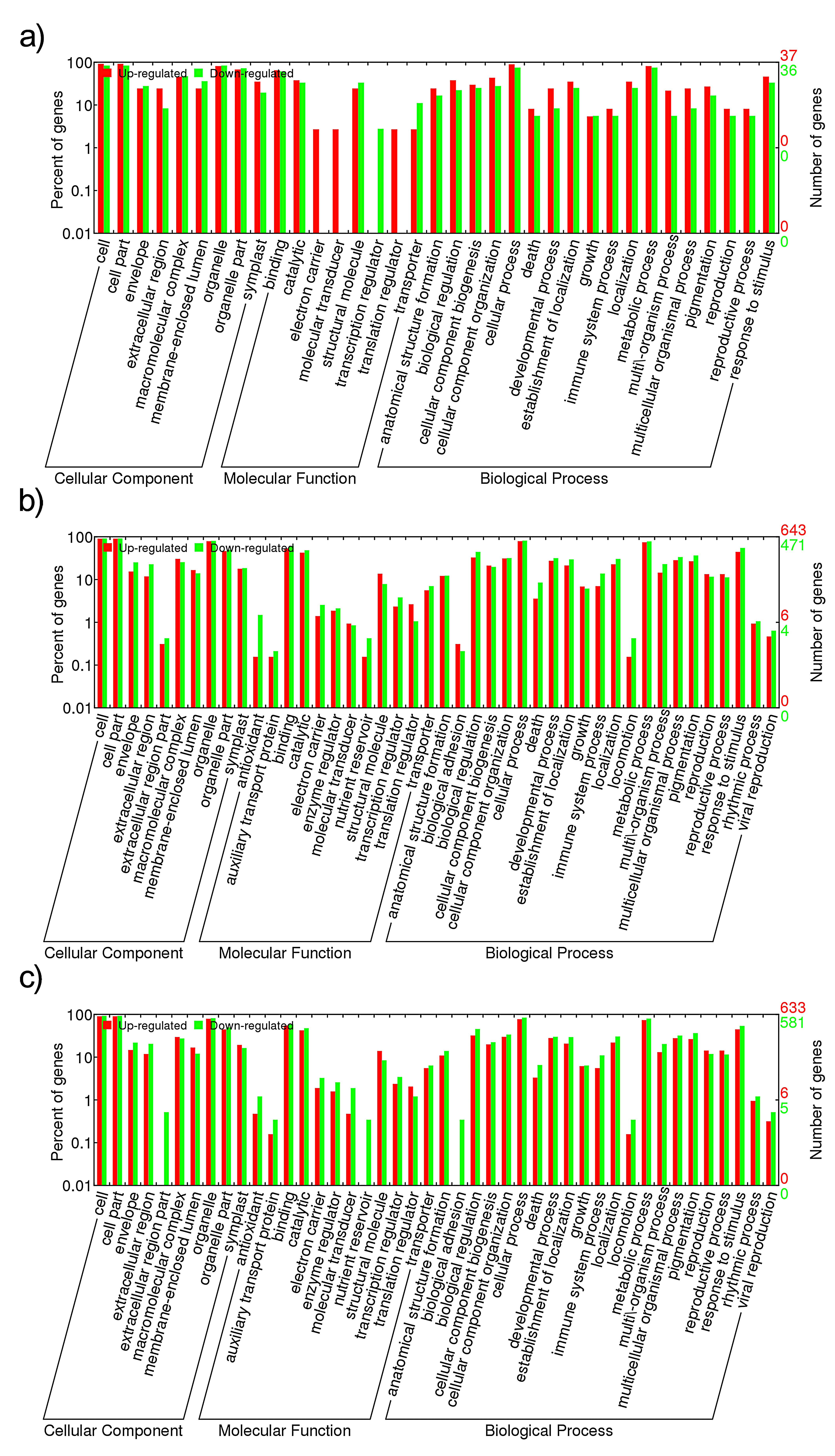

Supplement: S1 Fig — (TIF) [file pone.0169641.s001.tif]
